# Supplementary material for: JNK-Dependent cJun Phosphorylation Mitigates TGFβ- and EGF-Induced Pre-Malignant Breast Cancer Cell Invasion by Suppressing AP-1-Mediated Transcriptional Responses
Source: Cells. 2019 Nov 21;8(12):1481. doi: 10.3390/cells8121481 (PMC6952832; doi:10.3390/cells8121481)
Supplement: Supplementary file 1 [file cells-08-01481-s001.pdf]

Supplementary Figure S1

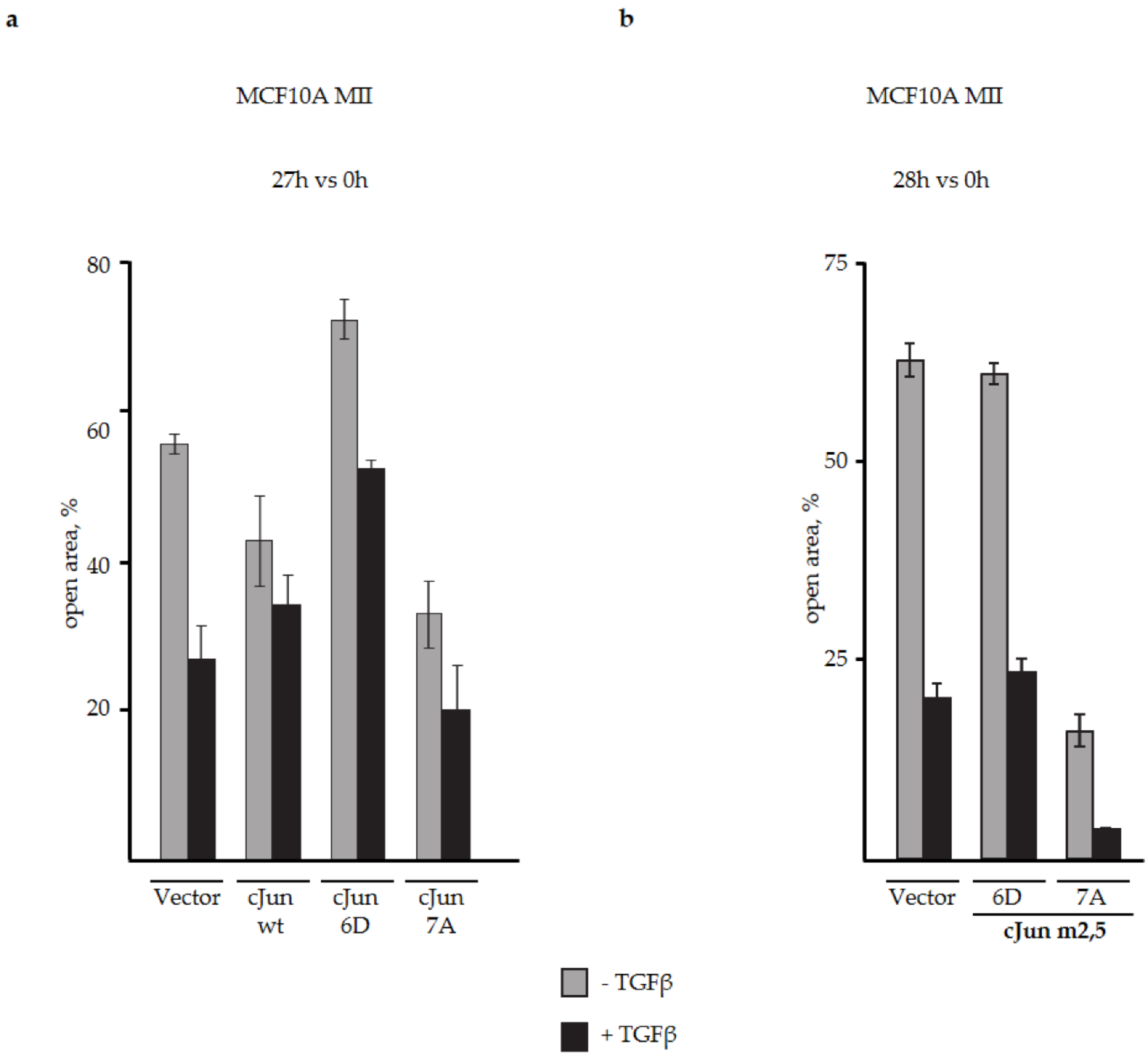

Supplementary Figure S1 JNK-dependent cJun phosphorylation negatively affects MCF10A MII cell migration. **(a)** Migration of MCF10A MII cells stably overexpressing of wt cJun,and cJun-6D and cJun-7A mutants in the presence or absence of TGFβ (5 ng/ml) for 27 h, as measured by wound healing assays; **(b)**Migration of MCF10A MII cells stably overexpressing the HA-tagged Fos- (m2,5) preferring variants of cJun-6D and cJun-7A in the presence or absence of TGFβ (5 ng/ml) for 28 h, as measured by wound healing assays.

Supplementary Figure S2

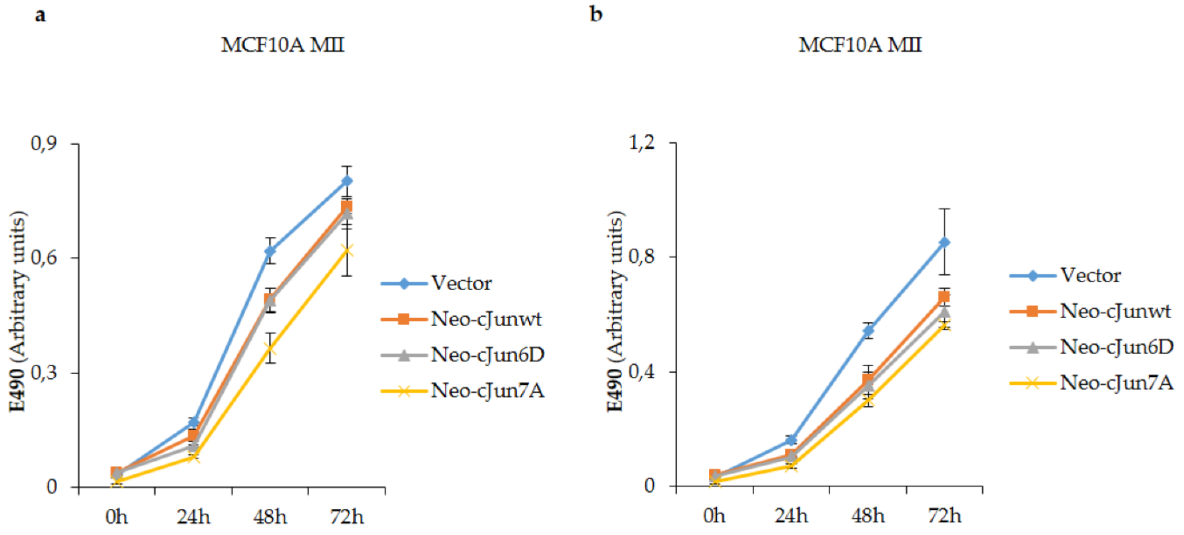

Supplementary Figure S2. MCF10A-MII cells expression ectopic cJun-7A did not show enhanced proliferation. **(a,b)** Proliferation of MCF10A-MII cells stably overexpressing wt cJun, cJun-6D, or cJun-7A, and the vector control, growing for the indicated times in complete media + 0,2%FBS (a), or in complete media + 0,2%FBS and 5ng/ml TGFβ (b). The assays were performed in triplicate using the CellTiter 96 AQ_{ueous} One Solution Cell Proliferation Assay Kit from Promega.

Supplementary Table S1. Primer sequences for qRT-PCR

Name	Sequence
FLNA FW	5'-CATCTGATGGACCAGCCTCTC-3'
FLNA Rev	5'-GCAGTTGGCTGTTGATCTGC-3'
GAPDH FW	5'-GGAGTCAACGGATTTGGTCGTA-3'
GAPDH Rev	5'-GGCAACAATATCCACTTTACCA-3'
HBEGF FW	5'-TCCTCTCGGTGCGGGACCAT-3'
HBEGF Rev	5'-GTGCCGAGAGAACTGCAGCCAG-3'
MMP1 FW	5′-CCAAATGGGCTTGAAGCT-3′
MMP1 Rev	5′-GTAGCACATTCTGTCCCTAA-3′
MMP2 FW	5'-AGATGCCTGGAATGCCAT-3'
MMP2 Rev	5'-GGTTCTCCAGCTTCAGGTAAT-3'
MMP10 FW	5'-CCCCTGGTGCCCACAAA-3'
MMP10 Rev	5'-TCACACTTGGCTGGCATCTC-3'
SERPINE1 FW	5'-GAGACAGGCAGCTCGGATTC-3'
SERPINE1 Rev	5'-GGCCTCCCAAAGTGCATTAC-3'
WNT7A FW	5'-TGCCCGGACTCTCATGAAC-3'
WNT7A Rev	5'-GTGTGGTCCAGCACGTCTTG-3'
WNT7B FW	5'-AAGCTCGGAGCACTGTCATC-3'
WNT7B Rev	5'-ACTGGTACTGGCACTCGTTG-3'
